# Anti-inflammatory effect of *Fumaria parviflora* leaves based on TNF-α, IL-1, IL-6 and antioxidant potential

**Published:** 2017

**Authors:** Waseem Rizvi, Mohammad Fayazuddin, Ompal Singh, Shariq Naeem Syed, Shagufta Moin, Kafil Akhtar, Anil Kumar

**Affiliations:** 1 *Department of Pharmacology, Jawaharlal Nehru Medical College, Aligarh Muslim University, Aligarh 202002, India*; 2 *Department of Pharmacology, Raichur Institute of Medical Sciences, Raichur, 584101, India*; 3 *Chemical Research Unit, Department of research in Unani Medicine, Aligarh Muslim University, Aligarh 202002, India*; 4 *Department of Biochemistry, Jawaharlal Nehru Medical College, Aligarh Muslim University, Aligarh 202002, India*; 5 *Department of Pathology, Jawaharlal Nehru Medical College, Aligarh Muslim University, Aligarh 202002, India*

**Keywords:** Fumeria parviflora, Carrageenan, Cytokines, Antioxidant, Anti-inflammatory

## Abstract

**Objective:**

In this study, we evaluated anti-inflammatory activity of leaves of *Fumaria parviflora *(*F. parviflora*) and underlying mechanisms by using *in vivo* models of inflammation.

**Material and Methods:**

Albino Wistar rats of either sex weighing 150 - 200 g were used. Soxhlet ethanol and aqueous extracts of leaves of *F. parviflora* (EFP and AFP) were prepared. The anti-inflammatory activity was studied using carrageenan-induced paw edema method and cotton pellet granuloma method. Levels of cytokines such as TNF-α, IL-6 and IL-1 and activity of antioxidant enzymes including catalase (CAT) and glutathione peroxidase (GPx) were estimated.

**Results:**

Leaves of *F. parviflora* demonstrated significant (p<0.001) decrease in paw edema in carrageenan-induced paw edema method. It diminished the serum tumour necrosis factor-α (TNF-α), IL-6 and IL-1 levels and also significantly attenuated the malondialdehyde (MDA) levels. The activity of CAT and GPx was increased in paw tissue. It also demonstrated significant decrease in granuloma formation in cotton pellet-induced granuloma method.

**Conclusion:**

Leaves of *F. parviflora* possess anti-inflammatory activity as they inhibit various cytokines and have antioxidant effects and free radical scavenging activity.

## Introduction

*Fumaria parviflora *Lam. (Fumariaceae) an annual herbaceous plant that grows in various parts of the Indo-Pakistan subcontinent, Middle East and South Asia. It is commonly known as fine leaf fumitory, Indian fumitory or wax dolls in English (Orhan et al., 2010 ▶). It has been traditionally used in Greco-Arab (Unani) traditional medicine and Iranian folk medicine for liver, bile duct and gut disorders, dermatological diseases such as acne, eczema and scabies and as a diuretic, antipyretic, expectorant, diaphoretic and antineoplastic agent (Amin, 1991 ▶; Zargari, 1989 ▶). It has also been traditionally used in folk medicine for promoting male fertility(Amin, 1991 ▶) *F. parviflora* is reported to have hepatoprotective, antioxidative (Jamshidzadeh and Niknahad, 2006 ▶) and antinociceptive activity (Heidari et al., 2004 ▶; Rao et al., 2007 ▶) and has a beneficial effect against hand eczema in humans (Jowkar et al., 2011 ▶). It has also demonstrated a protective effect against paracetamol-induced hepatotoxicity (Gilani et al., 1996 ▶).*F. parviflora *has shown a favorable effect on spermatogenesis in male rats (Heydari et al., 2012 ▶).However, to our best knowledge, there are no studies reporting the anti-inflammatory activity of leaves of *F. parviflora*. Hence, this study was conducted for evaluation of anti-inflammatory activity of leaves of *F. parviflora* and the underlying mechanisms using *in vivo* acute and sub-acute models of inflammation. Two extracts (aqueous and ethanolic) that were selected for the study contain different phytochemical constituents which have free radical scavenging activity that may have a possible role in countering the inflammatory response. The aqueous extract mainly contains the flavanoids (flavonols and quercetin) whereas the ethanolic extract possesses alkaloids (like fumariline, dihydrofumariline, fumaritine, and oxyberberine) and phenols (vanillicacid and cis-and trans-isomers of ferulic acid) (Gohil& Daniel, 2014 ▶ ; Hamedeyazdan, 2012 ▶) 

## Materials and Methods

**Plant material and extraction**

The leaves of *F. parviflora* were collected from local fields from Aligarh in March 2012,shade-dried, identified and authenticated by Prof Wajahat Ali, Department of Botany, Aligarh Muslim University and a voucher specimen was submitted (Rump 109). Dried leaves were powdered using a mechanical grinder and 100 g of the powder was extracted either with 300 ml of distilled water or ethanol for aqueous and ethanolic extract, respectively using Soxhlet apparatus. The extracts were collected in Petri dishes and evaporated to dryness in an incubator. The yield was 10.24% and 14.30% for aqueous and ethanolic extract, respectively. Then, the extracts were sealed with aluminum foil and stored at 4ºC for further experimental work. 

**Drugs and chemicals**

Aspirin (Reckitt Benckiser, India), propylene glycol (BDH, Mumbai) and carrageenan (Sigma Chemicals, USA) were used in the study. The other chemicals used were of analytical grades manufactured by Merck Laboratories (Mumbai, India). Also, Rat TNF-α Elisa Kit (Biomolecular Integrations), Rat IL-6 Elisa Kit (Koma Biotech, Korea), and Rat IL-1α Elisa Kit (Boster Biological Technology, LTD) were obtained.

**Animals**

Albino Wistar rats of either sex weighing (150 - 200 g) were procured from the Central Animal House, JNMC, Aligarh Muslim University. They were housed in polypropylene cages at ambient temperature (25 ± 2ºC) with relative humidity (55 ± 5%) and 12hr-12 hr light-dark cycle. Animals had access to standard pellet diet and water, *ad libitum*. The study protocol was approved by the Institutional Animal Ethics Committee (RegNo.401/CPCSEA).

**Experimental design**

Animals were divided into six groups of six animals each. 

1. Group 1(Control): Animals received normal saline 2 ml/kg p.o.

2. Group 2(Standard): Animals received and was given aspirin (100 mg/kg) p.o.

3. Groups 3 and 4: Animals received ethanolic extract of *F. parviflora* (EFP) at the dose of 300 and 500 mg/kg p.o., respectively.

4. Groups 5 and 6: Animals received aqueous extract of *F. parviflora* (AFP) at the dose of 300 and 500 mg/kg p.o., respectively.

**Carrageenan-induced paw oedema method**

This is one of the most commonly employed methods for the screening of acute inflammation. All the groups were treated with single dose of respective drug and 1 hr after the administration of the drugs, acute inflammation was produced by sub-plantar injection of 0.1 ml of freshly prepared suspension of 1%carrageenan in normal saline, to the right hind paw of the rats (Winter et al., 1962 ▶). The paw was marked at the level of the lateral malleolus and immersed every time up to this mark. The paw volumes were measured at 0hr, 1hr, 2hr, and 3hr after the carrageenan injection using digital plethysmometer (Orchid scientific, India).

The percentage inhibition of paw edema at each time interval was calculated by using the following formula:

Percentage inhibition=Vt-Vocontrol-Vt-Vo treatedVt-Vo control×100

Where, Vo =Paw volume of test/control group at 0 hr

Vt= Paw volume of test/control group at that particular time interval.

At the end of experiment, rats were anesthetized and right hind paw tissue was taken and homogenized with cold phosphate buffer solution (4x). Then, the homogenate was centrifuged and supernatant was obtained and stored at −20 ºC for MDA and antioxidant enzyme activity assays. 

**Cotton pellet-induced granuloma method**

This method is commonly used for the screening of sub-acute inflammation. Under anaesthesia with aseptic precautions, two sterilized cotton pellet (10mg) were implanted subcutaneously in either side of lumbar region in each rat. The incisions were sutured by silk 2.0 sutures and the wounds were sealed with betadine solution to prevent contamination. Bleeding was minimal and the animals recovered within 5 - 10 min after the anaesthesia. All groups received the treatments daily for 7 days including the day of implantation of pellets. On the eighth day, the animals were anaesthetized with Ether, the cotton pellets were removed and dried at 60 ºC for 24 hr. The dry weight of the granuloma was calculated by recording the difference in the dry weight of the cotton pellets before and after implantation (Winter & Porter, 1957 ▶).

Percentage of inhibition was calculated by using the following formula:

$\mathrm{Percentage of inhibition}=\frac{\mathrm{WC}-\mathrm{WT}}{\mathrm{WC}}\times 100$

Where, WC = Weight of the cotton pellets in control animal.

WT = Weight of the cotton pellets in drug treated animals.

**Estimation of TNF-α, IL-6 and IL-1 levels in serum**

After completion of the carrageenan-induced paw edema experiment, rats were anesthetized and blood samples were collected from orbital sinus. The serum was separated by allowing blood to clot followed by centrifugation and the samples were stored at −20ºC until use. TNF-α, IL-6 and IL-1 levels from each sample were measured in duplicate with highly sensitive rat TNFα Elisa kit (Biomolecular Integrations), rat IL-6 Elisa kit (Koma Biotech), rat IL-1α Elisa kit (Boster Biological Technology LTD) respectively, specifically designed for rats, according to manufacturer’s instructions.

**Determination of tissue lipid peroxidation**

Malondialdehyde (MDA) from carrageenan-induced edema foot was evaluated by the thiobarbituric acid reacting substance (TRARS) method (Ohishi et al., 1985 ▶). Briefly, at high temperature, MDA reacted with thiobarbituric acid at acidic pH resulting in formation of a red complex TBARS. The absorbance of TBARS was determined at 532 nm.

**Determination of antioxidant enzyme activity**

The GPx enzyme, in the presence of H_2_O_2_, reduces H_2_O_2_ to water by catalyzing the reaction of GSH turning into oxidized glutathione (GSSG). The resulting GSSG is reduced again to GSH by the glutathione reductase reaction using NADPH as the reducing substrate. There is a decrease in absorbance during the oxidation of NADPH to NADP^+^ and this is measured by a spectrophotometer at 340 nm for the calculation of the GPx activity (Paglia and Valentine, 1967 ▶). The results were given as U/mg protein. Total catalase (CAT) activity estimation was done as follows. The reduction of 10 mM H_2_O_2_by 20mM of phosphate buffer (pH 7) was monitored by measuring the absorbance at 240 nm(Armstrong & Browne, 1994 ▶). The activity was calculated by using a molar absorption coefficient, and the enzyme activity was defined as nano moles of dissipating hydrogen peroxide per milligram protein per minute. Protein concentration was measured by Lowry method (Lowry et al., 1951 ▶) using bovine serum as a standard. The enzyme activities were expressed as units of enzyme activity per milligram of protein.

**Histological examination**

Under deep anaesthesia, biopsies of the paws were taken 3hrafter the injection of carrageenan. The tissue slices were fixed in 10% neutral-buffered formaldehyde, dehydrated by graded ethanol and embedded in paraffin. Then, 5μm thick slices were sectioned and stained with hematoxylin and eosin. All samples were observed and photographed using Olympus microscopy. Tissue slices were randomly chosen from carrageenan, aspirin, EPF and APF-treated groups. The numbers of neutrophils were counted in each field (400x) and average count from 5 fields of every tissue slice was calculated.

**Statistical analysis**

All the values are expressed as mean ± SEM (n=6). Statistical significance was measured by one way ANOVA followed by *post-hoc *Dunnett’s multiple comparison test. p<0.05 was considered to be statistically significant.

## Results

**Effect of ethanol and aqueous extract on paw edema**

**Figure 1.**
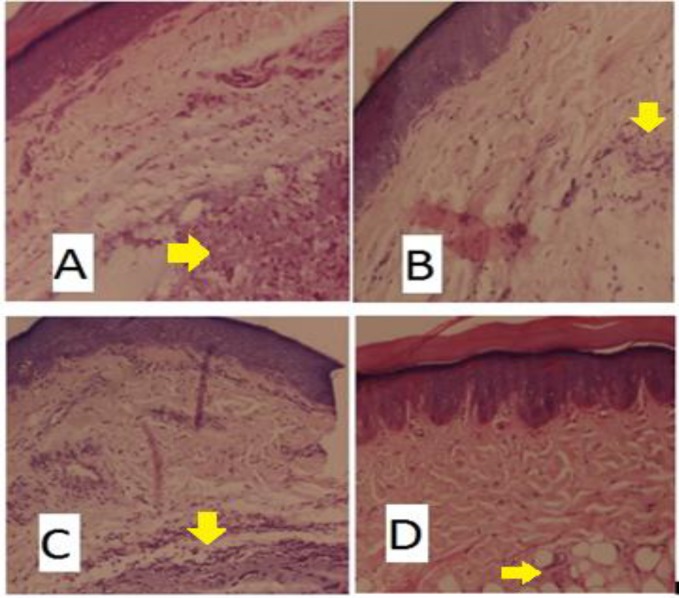
Histological examination of paw sections 3 hr after carrageenan injection. (a) Control, (b) aspirin group, (c) aqueous extract group(500mg/kg),(d) ethanolic extract group(500mg/kg) at 400X

**Figure 2:**
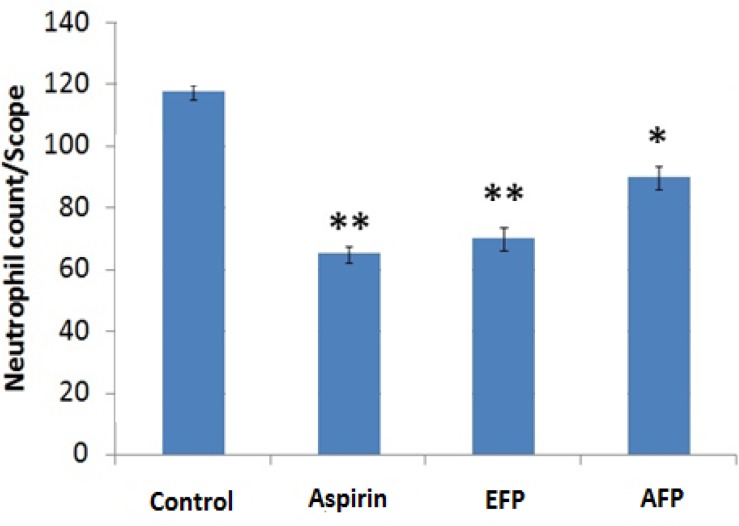
Effect of *F. parviflora* leaf extract on neutrophil infiltration in rat paw after carrageenan injection. Values are expressed as mean ± standard error of the mean. *p< 0.05, ** p< 0.001 when compared to the control group. EFP 500mg/kg; AFP 500mg/kg.

As shown in Table 1, aspirin (100 mg/kg) significantly decreased the paw edema 1hr (p<0.001) and 3hr (p<0.001) after carrageenan injection compared to control and percentage of inhibition of edema was 59.58% and 87.06% at 1and 3hr, respectively. Both EFP and AFP-treated groups demonstrated dose-dependent decrease in paw edema compared to control group. EPF showed significant reduction in paw edema both at 1hr and 3hr after carrageenan injection compared to control and percentage of inhibition of edema was 34.18% (p<0.05) and 36.11% (p<0.001)at 1and 3hr, respectively with EPF 300mg/kg and was 38.30% (p<0.001) and 71.77% (p<0.001)at 1and 3hr, respectively with EPF 500mg/kg. APF also showed significant reduction in paw edema both at 1hr and 3hr after carrageenan injection compared to control and percentage of inhibition of edema was 17.30% and 30.59% (p<0.001) at 1 and 3hr, respectively with APF 300mg/kg and was 27.66% (p<0.01) and 50.59% (p<0.001)at 1and 3hr, respectively with APF 500mg/kg. Histological examination of paw sections of rats treated with carrageenan revealed a significant tissue injury accumulation of edema fluid (Figure 1A) and increased infiltration of neutrophils (Figure 2). Groups treated with *F. parviflora *extracts showed a reduction in carrageenan-induced inflammatory response and there was significant decrease in the number of neutrophils as compared to the carrageenan-treated group (p< 0.001) (Figure 1C,1D and2).

**Table 1:** Effect of leaves of *Fumaria* *parviflora* on carrageenan-induced paw edema

**Groups**	**Paw volume at different time interval (ml)**				**% Inhibition of edema**	
	**0 hr**	**1 hr**	**2hr**	**3hr**	**1hr %**	**3hr%**
**Control**	0.80+.37	1.27±.046	1.40±.045	1.65±.058	-	-
**Aspirin (100 mg/kg)**	0.82±.035	1.01±.026**	1.04±.043**	0.93±.035**	59.58	87.06
**EFP (300 mg/kg)**	0.85±.021	1.15±.30**	1.20±0.38**	1.14±.047**	36.18	34.11
**EFP (500 mg/kg)**	0.77±.029	1.06±.032**	1.07±.040**	1.01±.037**	38.3	71.77
**AFP (300 mg/kg)**	0.88±.035	1.27±.040	1.35±.043**	1.43±.039**	17.03	30.59
**AFP (500 mg/kg)**	0.82±.029	1.16±.052**	1.19±.029**	1.24±.032**	27.66	50.60

Values are expressed as mean±S.E.M.

* p<0.05

** p <0.001 when compared to the control group; EFP:Ethanolic extract of leaves of *F. parviflora*; AFP: Aqueous extract of leaves of *F. parviflora.*

**Effect of ethanolic and aqueous extract on granuloma**

**Figure 3:**
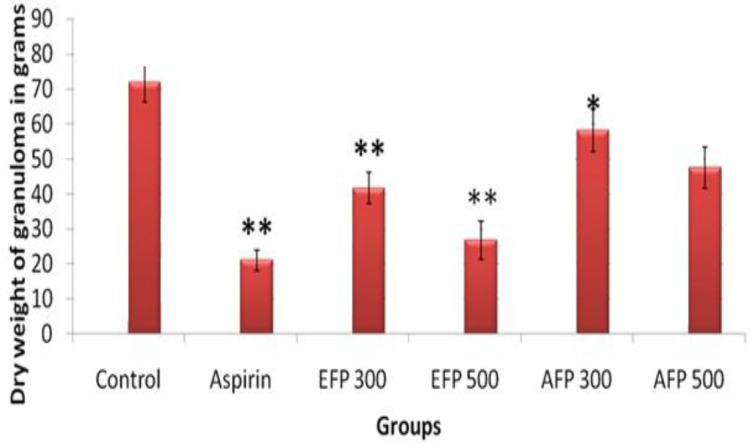
Effect of *F. parviflora* leaf extract on dry weight of cotton pellet granuloma.

In cotton pellet-induced granuloma method, aspirin 100 mg/kg significantly inhibited the granuloma formation by 70.68% (p<0.001) when compared to control. Whereas EPF significantly inhibited the granuloma formation by 41.96% (p<0.001) and 62.68% (p<0.001) compared to control group, at the doses of 300mg/kg and 500mg/kg, respectively. APF significantly decreased granuloma formation by 19.03% (p<0.05) and 33.89% (p<0.001) at the doses of 300mg/kg and 500mg/kg, respectively when compared to control (Figure 3).

Values are expressed as mean ± standard error of the mean. *p< 0.05, ** p< 0.001 when compared to the control group. EFP (ethanolic extract of *F. parviflora* leaves) 500mg/kg; AFP (aqueous extract of *F. parviflora* leaves) 500mg/kg.

**Effect of ethanolic and aqueous extract on level of serum cytokines**

As shown in Table 2, there was a dose-dependent decrease in all the three serum cytokines (TNF, IL-6 andIL-1) in EPF-treated groups at both 300mg/kg (p<0.05) and 500mg/kg (p<0.001) doses whereas in APF-treated groups significant decrease was seen only at 500mg/kg (p<0.05). 

**Table 2:** Effects of leaves of *Fumaria* *parviflora* on serum cytokines

**Groups**	**TNF-α(pg/ml)**	**IL- 6** **(pg/ml)**	**IL- 1** **(pg/ml)**
**Control** **(1 ml/kg)**	707.50±65	605.25±43	252.50±26
**Aspirin** **(100 mg/kg)**	400.25±48**	282.00±33**	124.00±13**
**EFP** **(300 mg/kg)**	504.50±39**	469.75±33*	180.00±12**
**EFP ** **(500 mg/kg)**	451.50±21**	415.25±28**	165.24±12**
**AFP ** **(300 mg/kg)**	602.34±41	522.73±36	211.86±9
**AFP ** **(500 mg/kg)**	552.56±26*	480.25±33*	191.25±11*

Values are expressed as mean±S.E.M.

* p<0.05

** p <0.001 when compared to the control group. EFP: Ethanolic extract of leaves of *F. parviflora*, AFP: Aqueous extract of leaves of *F. parviflora*.

**Effect of ethanolic and aqueous extract on enzymes activity**

Also, there was a significant dose-dependent decrease in MDA levels in both EPF (p<0.001) and APF (p<0.001)-treated groups and also there was a significant dose-dependent increase in catalase and GPx activities in all the groups treated with EPF (p<0.001) and APF (p<0.001) (Table 3).

**Table 3:** Effects of leaves of *Fumaria* *parviflora* on malondialdehyde, catalase and glutathione peroxidase

**Groups**	**MDA(nmol/** **mg protein)**	**Catalase ** **(U/** **mg protein)**	**GPx** **(U/** **mg protein)**
**Control** **(1ml/kg)**	1.60±.08	3.22±.05	9.60±.27
**Aspirin ** **(100 mg/kg)**	0.74±.03**	4.60±.11**	16.72±.18**
**EFP (300 mg/kg)**	1.25±.04**	4.10±.03**	12.98±.09**
**EFP (500 mg/kg)**	1.08±.04**	4.31±.10**	14.86±.13**
**AFP (300 mg/kg)**	1.40±.03**	3.51±.06**	10.58±.24**
**AFP (500 mg/kg)**	1.32±.02**	3.93±.07**	11.22±.25**

Values are expressed as mean±S.E.M.

* p<0.05,

** p<0.001 when compared to the control group EFP: Ethanolic extract of leaves of *F. parviflora*, AFP: Aqueous extract of leaves of *F .parviflora*.

## Discussion

The carrageenan-induced edema in rat hind paw is the most widely-used primary test for screening anti-inflammatory agents. Its molecular mechanism is well characterized, and is considered as as standard model of inflammation for screening anti-inflammatory activity of different compounds. The development of edema after injection of carrageenan is a biphasic event. The initial phase of inflammation which is observed during the first hour, is attributed to the production of histamine, leukotrienes, and possibly cyclooxygenase products, while the delayed phase of the carrageenan-induced inflammatory response has been linked to neutrophil infiltration and the production of neutrophil-derived free radicals, such as hydrogen peroxide, superoxide, and OH radicals, as well as the release of other neutrophil-derived mediators (Vinegar et al., 1969 ▶). However, a decrease in paw swelling volume is a good index in determining the protective effect of anti-inflammatory agents. In the present study, ethanolic extract of *F. parviflora *showed significant reduction of edema in both phases of inflammation but maximum reduction was observed in the second phase of inflammation (62.36%). Aqueous extract showed significant (p<0.01) reduction of edema mainly in the second phase of inflammation. The effect of *F. parviflora *lasted for 3 hours comparable to that of aspirin. This anti-inflammatory activity can be attributed to various phytochemicals like isoquinoline alkaloids, flavonoids, phenolic compounds and glycosides present in *F. parviflora* (Naz et al., 2013 ▶, Sousek et al, 1999 ▶). Flavonoids are known to inhibit the synthesis of prostaglandins which are involved in acute inflammation (Raj et al., 2001 ▶). Phytochemical analysis of *F. parviflora* leaves has shown that they are also rich in alkaloids and phenolic compounds which have been reported to possess antioxidant activity by decreasing various mediators of inflammation like prostaglandins, NO, TNF-α, IL-6 and IL-1(Lee et al., 2009 ▶). TNF-α is an important inflammatory mediator which induces immune response by activating macrophages and T cells and stimulating secretion of various other inflammatory cytokines such as IL-6 and IL-1 (Liao et al., 2011 ▶). TNF-α is also involved in carrageenan-induced inflammatory response and induces further release of kinins and leukotrienes, which are thought to play an important role during inflammatory response (Dawson et al., 1991 ▶). In this study, we found that *F. parviflora *decreased the levels of TNF-α IL-6 and IL-1 in serum after carrageenan injection. Also, there is neutrophil infiltration and generation of neutrophils-derived free radicals such as hydroxyl radicals, hydrogen peroxide and superoxide and release of other neutrophil-derived mediators in carrageenan-induced inflammatory response. In the present study, the histopathology revealed marked decrease in the cellular infiltration by neutrophils and tissue damage. Lipid peroxidation is due to the attack of free radicals to lipids in cell membranes resulting in accumulation of MDA and inflammation. Lipid peroxidation not only serves as a marker of tissue damage *in vivo *but also has been recognized as an inducer of inflammatory processes (Chaturvedi, 2008 ▶). In this study, leaves of *F. parviflora *not only exhibited radical scavenging capacity but also decreased carrageenan-induced lipid peroxidation. Excess reactive oxygen species (ROS) tend to cause oxidative imbalance of the antioxidant system which may result in oxidative stress and inflammation. Given the importance of the oxidative status in the formation of edema, the anti-inflammatory effect exhibited by the extract in this model might be related to its antioxidant properties (Bignotto et al., 2009 ▶). The antioxidant enzymes, catalase and GPx play crucial role in scavenging H_2_O_2_ and hydroperoxide (Huang et al., 2012 ▶). In this study, there was a significant increase in catalase and GPx activities and anti-inflammatory effect of *F. parviflora *could be due to elevated intracellular antioxidant enzyme activities and decreased oxidative stress in tissues. The cotton pellet granuloma method has been widely employed to study various components of sub-acute inflammation such as transudative, exudative and proliferative phases. The change in dry weight of the granuloma measures the proliferative phase due to monocyte infiltration and fibroblast proliferation that take place in chronic inflammation. (Swingle and Shideman, 1972 ▶) In this study, both ethanolic and aqueous extracts of *F.parviflora *significantly decreased the dry weight of the granuloma when compared to the control group. The percentage of inhibition for the ethanolic and aqueous extracts was highest at the dose of 500mg/kg (i.e. 62.68% and 33.89%, respectively) (p<0.001) (Figure 1). This anti-inflammatory action may be due to the ability of *F. parviflora *leaves in reducing the number of fibroblasts and synthesis of collagen and mucopolysaccharide, which are natural proliferative agents of granulation tissue formation. The inhibition of production of proinflammatory cytokines, such as IL-1, IL-6 and TNF-α which are powerful chemotactic agents for macrophages and fibroblasts as seen in this study, may be responsible for the anti-inflammatory effect of the extract as discussed earlier (Chang et al., 2011 ▶).

Our study suggests that leaves of *F. parviflora* possess anti-inflammatory effect which might be attributed to the inhibition of various cytokines, their antioxidant effects due to increase in the activities of antioxidant enzymes and their free radical scavenging effect.
